# Improving Semantic Interoperability in Health Care Through Translation and Contextualization of International Organization for Standardization 13940 (ContSys) in Estonia: Qualitative Document- and Artifact-Based Study

**DOI:** 10.2196/81227

**Published:** 2026-06-29

**Authors:** Kristian Juha Ismo Kankainen, Janek Metsallik, Ruth Erm, Gunnar Piho, Peeter Ross

**Affiliations:** 1 Department of Health Technologies School of Information Technologies Tallinn University of Technology Tallinn Estonia; 2 Medical Terminology Competence Center National Institute for Health Development Tallinn Estonia; 3 Department of Software Science School of Information Technologies Tallinn University of Technology Tallinn Estonia

**Keywords:** International Organization for Standardization 13940, ISO 13940, System of Concepts to Support Continuity of Care, ContSys, semantic interoperability, conceptual interoperability, translation, contextualization, continuity of care, event-based exchange, governance, reference architecture

## Abstract

**Background:**

Event-based digital health data and information exchange are a complex sociotechnical challenge because they rely on the existence of stable, shared meanings for care process concepts such as mandate, responsibility, episode boundaries, and referral, across clinical, administrative, financing, and technical stakeholders. International Organization for Standardization 13940:2015 System of Concepts to Support Continuity of Care (ContSys) provides a conceptual framework for continuity-of-care processes, but national translations and contextualization, along with their governance implications, remain largely undocumented in the scholarly literature.

**Objective:**

This study aimed to document Estonia’s translation and contextualization of ContSys and to identify and interpret recurring patterns of conceptual discrepancy that are relevant to the durable governance of event meanings.

**Methods:**

We conducted a qualitative study of a national standards implementation project by using document and artifact analysis. Materials included the source standard, the translated manuscript, mapping notes and spreadsheets, review records, and public commentary inputs. We preserved ContSys concepts while documenting local counterparts through country-specific notes (including scope differences and contextual legal use) and synonymous terms. We summarized implementation outputs by concept domain and interpreted discrepancy patterns by using the Levels of Conceptual Interoperability Model and Blobel’s Generic Component Model as a cross-domain reference architecture lens.

**Results:**

The Estonian publication covers all ContSys concept domains and includes extensive contextualization outputs (46 country-specific notes and 82 synonyms), with the highest concentrations in time- and responsibility-related domains, indicating where semantic pressure is the greatest. Mapping and review discussions repeatedly revealed conflation of local legal or organizational terms with distinct ContSys concepts, especially where mandate and responsibility shift over time. A familiar referral artifact label (Estonian: *saatekiri*) was inconsistently interpreted as (1) a mandate transfer, (2) joint involvement while retaining the original mandate, or (3) episode initiation, demonstrating why event meanings cannot be safely encoded as event triggers in specifications without an explicit, versioned meaning decision.

**Conclusions:**

Translating and contextualizing a conceptual standard can support cross-domain semantic alignment by making mismatches explicit while preserving conceptual fidelity. However, durable event meanings require an explicit stewardship model—decision rights, resourcing, conflict resolution, and change control—to maintain coherence as they evolve.

## Introduction

### Context: Event-Based Exchange and the Need for Stable Meanings

Digital health programs increasingly explore a shift from document-centric data and information exchange toward more event-oriented specifications. In practice, implementation guide ecosystems and platform programs push stakeholders to specify which care process events matter and under what mandate and responsibility, rather than focusing solely on how information is transported [1,2]. European Union cross-border exchange guidance further reinforces the need for consistent, shared event meanings across jurisdictions [3,4].

Continuity and coordination of care are widely treated as prerequisite capabilities for integrated, person-centered services, but they are operationally fragile unless responsibilities and handover semantics are explicit and shared across settings [5-7].

This creates a practical question that precedes technical “event-based” architectures: can stakeholders agree on stable event meanings in the first place? Familiar care process artifact labels (eg, “referral”) can encode incompatible assumptions about mandate, responsibility, and episode boundaries across domains—an ambiguity that must be resolved before being encoded into requirements and specifications.

Estonia’s Digital Agenda 2030 and the health sector’s next-generation exchange program identify a shift toward proactive, life- and business-event–based services as a desired future state, accompanied by renewed interoperability principles that emphasize semantic coherence across domains. This strategic direction implicitly requires that core care process events have stable, cross-domain meanings [2,8].

Scaling the nationwide Estonian Health Information System, which has been up and running since 2008, toward cross-domain continuity and multipurpose data use continues to expose semantic and organizational gaps—unclear roles, responsibilities, and decision rights; fragmented governance; cross-silo stewardship constraints; and documentation and data quality variation that limits reuse—even in a mature digital health setting [9-14]. These observed gaps represent current constraints on cross-domain interoperability.

We define event-meaning governance as a cross-domain capability for establishing and maintaining stable event meanings [15,16]. It includes recording decisions in artifacts that stakeholders can reference and version over time. In practice, this means keeping policy, payer rules, and implementation guidance aligned with the same governed meaning as they evolve.

A common concept system can help expose semantic mismatches across stakeholders and domains but does not by itself constitute authoritative event-meaning decisions. More generally, mandate and responsibility movement over time must be made explicit and governed before being encoded into event-based requirements and specifications [17,18].

### Conceptual Standards as Semantic Infrastructure

International Organization for Standardization (ISO) 13940:2015 System of Concepts to Support Continuity of Care (ContSys) provides a conceptual framework for describing continuity-of-care processes and the informational content within their process context [18]. The concept system consolidates earlier European prestandard work (ENV 13940) and has been positioned since the early 2000s as foundational conceptual infrastructure for continuity-of-care semantics, rather than as an implementation guide [19-24]. Early national translation and discussion efforts (eg, the National Board of Health and Welfare’s (Swedish: *Socialstyrelsen*) translation work on ENV 13940) illustrate that contextualization and governance questions have long accompanied the standard [25,26]. We treat ContSys as a cross-domain conceptual infrastructure: a shared concept system that helps clinical, administrative, financing, and technical stakeholders express and align care process event meanings (eg, mandate, responsibility, and episode boundaries) across artifacts, such as legislation, reimbursement rules, and implementation guidance. Conceptually, this aims to shift cross-domain interoperability work from multiple parallel, inductively emerging “local standards” toward governed alignment: using an explicit conceptual grammar to formulate compatible event meanings that can be carried coherently into laws, policies, and technical specifications.

This positioning also reflects a recurrent lesson in personalized health ecosystem work: interoperability at the data-exchange level is necessary but not sufficient for advanced, cross-domain coordination because the decisive gaps often lie in shared conceptualization, responsibility semantics, and governed evolution [27-30].

A central challenge is governance rather than terminology alone. Even with a stable concept system, semantic interoperability depends on practical arrangements for institutionalizing and sustaining meanings over time—ownership, resourcing, conflict resolution, and change control—so that policy and regulation, standards, and implementations remain mutually coherent as they evolve [31-33].

Against this background, Estonia translated and contextualized ContSys as a national standard using mapping, country-specific notes, and structured stakeholder review to make local meanings explicit without redefining core concepts [34]. In parallel, ContSys concepts were operationalized to structure obligation movement over time in a legal source relevant to care collaboration [35]. This paper builds on these efforts to examine governance-relevant discrepancy patterns through 2 lenses: the Levels of Conceptual Interoperability Model (LCIM) [17,36,37] and cross-domain reference architecture thinking using Blobel’s Generic Component Model (GCM), operationalized in ISO 23903 [32,38].

### Problem Statement

Estonia’s transition toward event-based exchange raises a sociotechnical interoperability challenge: stakeholders must establish stable, shared meanings of care process events to define events consistently across clinical and administrative settings, yet these meanings often diverge across domains.

The most difficult issues were not purely linguistic but emerged during contextual mapping: local legislation and organizational documentation sometimes conflated distinct concepts, especially when mandate transfer and responsibility over time had to be explicitly documented.

A recurring illustration from stakeholder discussions concerned a familiar referral artifact label (Estonian: *saatekiri*), whose meaning varies across settings and stakeholders. Such ambiguity is manageable in person-to-person coordination but becomes high risk in event-oriented specifications, where events must be defined and interpreted consistently across clinical, administrative, financing, and technical stakeholders. Without stable event meanings, specification work may encode incompatible assumptions across domains.

### Research Questions

The research questions were as follows: (1) how does the ambiguity in a familiar referral artifact label surface across clinical work, organizational mandate, payer and entitlement rules, and health information exchange specifications? and (2) how can ContSys translation outputs, interpreted via the LCIM and GCM, support durable governance of this event’s meaning?

We use *saatekiri* as an anchoring case to make cross-domain contradictions observable and to connect translation outputs to governance-relevant discrepancy patterns. We interpret these discrepancies through LCIM and GCM, as specified in the Methods section.

### Aim and Objectives

The aim was to establish a shared conceptual infrastructure for event-based digital health by translating ContSys into Estonian and contextualizing it within local terminology and legislation, without redefining or adapting its core concepts.

The objectives were to translate ISO 13940:2015 (ContSys) into Estonian with conceptual fidelity; contextualize the concept system by documenting local counterparts, scope differences, and contextual use through country-specific notes and term synonyms; report transparent scope indicators and recurring discrepancy patterns relevant for defining and governing care process events; and interpret key discrepancy patterns using LCIM and GCM, operationalized in ISO 23903, to clarify where the main interoperability barriers lie and what these barriers imply for cross-domain governance and specification work.

## Methods

### Study Design and Reporting Approach

We report a qualitative, document- and artifact-based study of a national translation and contextualization implementation of a conceptual standard. The empirical material comprised the source standard; the translated manuscript; and implementation artifacts produced during mapping, review, and publication (eg, mapping spreadsheets, review records, and public commentary inputs). No patient-level or identifiable personal data were processed. Stakeholder contributions were captured as role-based professional feedback within the standardization process and analyzed in an aggregated form.

### Relation to Prior Publications

This manuscript consolidates and extends two related publications: (1) the Estonian ContSys translation and contextualization experience report [34] and (2) a legal-domain application of ContSys concepts to structure obligation movement over time [35]. This study emphasizes cross-domain discrepancy patterns and their governance implications for defining and sustaining care process event meanings, using the referral artifact label (*saatekiri*) as an anchoring case.

### Setting and Participating Entities

The work was conducted as a national standards implementation project involving cross-domain stakeholders with roles in (1) terminology and language stewardship, (2) health information governance and platform program stewardship, (3) payer or entitlement interpretation, (4) care provider quality management, and (5) implementation and architecture. Translation and contextual mapping activities were carried out within an academic standards-development collaboration, with publication through the national standardization process. The project started in October 2021; translation and mapping concluded in 2022; and the national standard was published in February 2023.

### Intervention Scope

The intervention scope was the national translation and contextual mapping of ContSys. The study focused on governance-relevant event meanings and on discrepancy patterns that matter when mandate and responsibility shift over time. It did not implement computable event definitions in operational systems or assess interface-level conformance to specific message standards or implementation guides. Therefore, we report scope indicators of implementation intensity and recurring conceptual discrepancy patterns, not downstream adoption or technical alignment outcomes.

### Implementation Blueprint and Deviations

We operationalized the translation and contextualization approach described in the earlier experience report [34] as a six-task blueprint: (1) structured concept inventory and term work, (2) full-text translation, (3) quality assurance, (4) continuous mapping and contextualization, (5) 2-round proofreading, and (6) public commentary and publication.

Deviations from the planned approach were as follows:

- Computer-assisted translation with a term base was planned, but translation was completed in a word processor.
- A formal back-translation was planned but not performed.
- Therefore, quality assurance relied on iterative expert review during translation and mapping and on 2 rounds of proofreading.

Public commentary inputs included perspectives from information system governance, payer interpretation, and care provider quality management.

### Data Sources

Data sources comprised (1) ISO 13940:2015, (2) the translated standard manuscript, and (3) project artifacts (spreadsheets, mapping notes, review records, and public commentary inputs). Key national sources referenced during mapping included consolidated legislation [39] and payer guidance on referrals [40]. In addition, we used a small set of recurring stakeholder illustrations (including *saatekiri*) recorded in project mapping notes to capture and compare working definitions, identify conflated ContSys concepts, and validate interpretations against real-world workflows. No patient-level data were processed. Stakeholder inputs were collected and recorded as professional, role-based feedback during standard implementation.

### Outcomes and Scope Indicators

Because the intervention was a translation and contextualization implementation, not an evaluation of operational adoption, we report transparent scope indicators that can be extracted from project artifacts to describe implementation intensity and the location of semantic pressure. These include translation coverage, counts and distributions of country-specific notes and term synonyms, and a qualitative description of stakeholder engagement modes.

### Analysis and Synthesis

First, we counted the number of country-specific notes added to the Estonian ContSys. The distribution effectively captured our experiences from stakeholder discussions—the most problematic conceptual domains were those related to time and responsibility. While our other results showed near-perfect conceptual conformance of responsibility concepts in the Law of Obligations Act [35], this conflict is possible because other health care–related regulations implement these obligations using a single black-box device, *saatekiri*.

We then conducted a qualitative analysis of documents and artifacts to summarize recurring conceptual discrepancies and implementation outputs. To align with the Introduction section’s framing (governance-relevant event meanings), we used two analytic lenses:

1. Conceptual interoperability lens—it distinguishes agreement on different levels of interoperability. We used Tolk’s LCIM to evaluate the transition from semantic interoperability (shared knowledge of labels), pragmatic interoperability (shared knowledge of contexts), dynamic interoperability (shared knowledge of effects over time), and conceptual interoperability (shared knowledge of aims and underlying assumptions). This lens explains why the apparent semantic conflict can still support information exchange at a pragmatically autonomous level [17].
2. Cross-domain governance lens—it relates discrepancy patterns to the coordination of policy and regulation, standards, and implementation work over time. Building on our results from a previous study [35], which placed ContSys-encoded obligations as a gold standard that serves as the business viewpoint across the health care system, we used Blobel’s GCM as a reference architecture lens to locate where *saatekiri*-type discrepancies originate (domain practice and responsibility semantics) vs where alignment is implemented (implementation guidance, specifications, and systems) and to emphasize that event meanings must be maintained coherently across policy, payer rules, and implementation guidance as they evolve [32,33].

LCIM guided the interpretation of discrepancy patterns. The mapping of the anchoring case to LCIM levels is reported in the Results section. Where relevant, we link observed discrepancy patterns to the legal-domain annotation work that operationalizes mandate and obligation movement concepts in a concrete legal source [35]. Discrepancy patterns were grouped by issue type (eg, scope differences, conflation of distinct concepts, and missing local counterparts). As a concrete coding rule for the anchoring case, we coded each *saatekiri* interpretation against the ContSys demand-for-care abstraction [18] and its specializations (referral, request, and demand for initial contact) to make demand-type ambiguity explicit before interpreting mandate and responsibility movement over time.

As an analytic synthesis, we interpreted why key discrepancy patterns matter for event-based exchange by relating them to (1) conceptual interoperability levels (LCIM) and (2) cross-domain integration and evolution considerations (GCM, operationalized in ISO 23903) [17,32,33].

### Resources and Sustainability

Parts of the work were supported by the Health Sense project (Norway Grants) [41]. Long-term sustainability depends on continued terminology stewardship, resourcing for cross-domain mapping, and governance for maintaining country-specific notes and mappings.

### Ethical Considerations

Ethics approval was not applicable for this study as it analyzed documents and implementation artifacts produced during a national standardization process (eg, the translated standard manuscript, mapping notes, review records, and public commentary inputs), alongside publicly available legal and guidance sources. No patient-level data and no identifiable personal data were collected or processed. Organizational names referenced in this manuscript are included solely to identify publicly available documents, standards, and program materials and do not represent data collected from organizational staff or study participants. Consent to participate was also not applicable for this study. Stakeholder contributions were provided as role-based professional feedback within the standardization process and are reported only in aggregate form, without individual-level quotations or identifiers.

## Results

### Implementation Outputs and Coverage

We completed a full Estonian translation of ISO 13940:2015 (ContSys) together with continuous contextual mapping. The translated national standard covers all concept domains and sections. Contextualization outputs comprised country-specific notes and term synonyms (Table 1), representing comprehensive, but not exhaustive, mapping to local terminology and legislation.

Domains related to responsibility and time show relatively high counts, consistent with the mandate- and responsibility-related ambiguity illustrated by *saatekiri* (Table 2).

Table 2 illustrates the functions of country-specific notes (Estonia-specific note [EE NOTE]) and synonym mapping as governance-friendly mechanisms for making local mismatches explicit while preserving the underlying ContSys concept system. The example highlights how the publication documents differences (eg, scope mismatch and contextual legal use) rather than smoothing them over.

**Table 1:** Distribution of explanatory notes and synonyms by System of Concepts to Support Continuity of Care (ContSys) concept domain (from the published Estonian ContSys standard).

ContSys concept domain	Standard notes (n=65), n (%)	Estonia-specific notes (n=46), n (%)	Synonyms (n=82), n (%)
Concepts and terms	1 (1.5)	3 (6.5)	0 (0)
Actors	4 (6.2)	9 (19.6)	9 (11)
Health care matters	14 (21.5)	3 (6.5)	16 (19.5)
Activities	14 (21.5)	4 (8.7)	15 (18.3)
Process	6 (9.2)	2 (4.3)	4 (4.9)
Planning	9 (13.8)	2 (4.3)	6 (7.3)
Time	3 (4.6)	9 (19.6)	16 (19.5)
Responsibility	8 (12.3)	7 (15.2)	10 (12.2)
Information management	6 (9.2)	7 (15.2)	6 (7.3)

**Table 2:** Structure of a translated System of Concepts to Support Continuity of Care (ContSys) concept entry illustrating the standardized elements reported in the Estonian ContSys publication (example based on the ContSys entry “subject of care” [Estonian: patsient]).

Concept element	Example content (English translation of the Estonian publication)
Concept domain	Health care actor
Preferred term	*Patsient* (subject of care)
Synonyms	*Abisaaja* (beneficiary), *ravialune* (person under treatment), and *andmesubjekt* (data subject)
Definition	A health care actor in the role of a person and someone who wants to receive, receives, or has received care in a health care system
International Organization for Standardization note	A fetus may be considered a subject of care if it receives or has received treatment
EE NOTE^a^	EE NOTE 1: although Estonian law defines a patient as a natural person who has expressed the wish to receive, or is receiving, a health care service, the Estonian publication uses *patsient* also for a person who has received care in the health care system. EE NOTE 2: in some contexts, other labels are used instead of *patsient* (eg, a medical certificate applicant); in personal data processing, the patient is referred to as a data subject
Examples	Treated patient, physiotherapy client, a person selected for population screening, a member of a diabetes group participating in health education, and a person seeking health advice
Associations	Linked to treatment, health state, health need, personal health record, self-care, next of kin, consent capacity, subject-of-care desire, informed consent, refusal of assistance, health care contact, proxy decision rights, and preference delay and linked to the fact that health care activities and processes, authorizations, health care matters, and health records are defined “for” or “concerning” the subject of care

^a^EE NOTE: Estonia-specific note.

### Stakeholder Participation and Review Footprint

Stakeholder engagement is combined with iterative expert reviews during translation and mapping, with an open public commentary phase. Participation covered the main cross-domain perspectives needed for contextualization (terminology or language stewardship, health information governance, payer or financing interpretation, care provider quality management, and implementation or architecture), but quantitative totals (eg, review rounds and participant counts) were not consistently captured (Table 3).

Participation varied across domains, and some intended contributions (eg, deeper reuse-oriented architectural mapping) were not realized due to resource constraints.

**Table 3:** Qualitative summary on stakeholder categories, typical roles, and engagement modes.

Stakeholder category	Typical roles	Engagement mode
Terminology and language stewardship	Terminologists and linguists	Workshops and document review
Health information governance	Stewards and program owners	Structured workshop and document review
Payer and financing perspective	Reimbursement specialists and claims interpretation	Structured workshop and public commentary
Care provider quality management	Quality leads and process owners	Public commentary and targeted review
Implementation and architecture	Architects and implementers	Targeted review

### Structure of the Estonian Publication and Documented Deviations

The Estonian publication mirrors the original standard structure (concept domains, preferred terms, synonyms, definitions, notes or examples, and concept associations). A key implementation decision for preserving conceptual fidelity while documenting local meaning was the explicit prefixing of country-specific notes with a national identifier (eg, “EE NOTE”), ensuring a clear separation between local contextualizations and the original standard.

### Recurring Conceptual Discrepancy Patterns

Across mapping and stakeholder discussions, we observed recurring discrepancy patterns:

- Scope differences—local legal or organizational definitions covered a narrower or broader set of cases than the corresponding ContSys concept.
- Conflation of distinct concepts—local sources used a single term for separate concepts in ContSys.
- Missing local counterparts—ContSys concepts lacked direct equivalents in local documentation, requiring explanatory notes.

A particularly critical pattern involved mandate-related distinctions, in which local documentation conflated concepts that ContSys distinguishes by responsibility and mandate transfer over time. Across these patterns, the dominant barriers were located above the interface level (as described by LCIM) and required cross-domain coordination of meaning decisions and their evolution over time (as described by GCM), rather than additional interface-level harmonization alone.

Illustrative example (referral label; *saatekiri*). In stakeholder discussions and mapping notes, the same referral label was interpreted in at least three incompatible ways: (1) mandate transfer (ending an existing mandate), (2) joint involvement (retaining the existing mandate while adding another actor or service), or (3) initiation of a new mandate or episode. To make the ambiguity explicit and comparable across domains, we anchored *saatekiri* under the ContSys abstraction “demand for care” and treated these readings as *candidate mappings* to its specializations: referral, request, or demand for initial contact [18].

The governance-relevant question is not whether a *saatekiri* artifact exists, but which demand-for-care specialization is intended and what responsibility and mandate movement follows from it. Table 4 summarizes the candidate mappings, the corresponding governance decisions, and the boundary conditions.

These gaps matter because event-oriented specifications require explicit, shared assumptions about who is responsible, when, and under what mandate—assumptions that must remain stable across clinical operations and payer interpretation. Table 5 maps the *saatekiri* findings to LCIM levels to clarify where the interoperability barrier sits and what must be governed before event meanings can be safely encoded in specifications.

Table 5 distinguishes agreement on exchange and formats from agreement on intent, effects over time, and underlying assumptions. It supports the claim that *saatekiri*-type ambiguity sits primarily at the pragmatic, dynamic, and conceptual levels and therefore requires event-meaning governance rather than interface harmonization alone.

The ambiguity was repeatedly observable because different stakeholder domains approach the same familiar label through different governing artifacts and accountabilities (clinical procedures, organizational mandate arrangements, entitlement and reimbursement rules, and specification guidance). When the label is treated as a single event trigger, these locally coherent readings become incompatible across domains—hence the need for an explicit, maintained event-meaning decision rather than ad hoc coordination.

In the Estonian publication, such conflations were made visible through country-specific notes that distinguish mandate transfer, joint involvement, and episode initiation. Notes document distinctions, but durable event meanings require maintained governance across policy, reimbursement, and implementation guidance, supported by implementation documentation and payer workflow descriptions.

**Table 4:** Candidate mappings of saatekiri to the System of Concepts to Support Continuity of Care (ContSys) demand-for-care specializations and the corresponding governance decision.

Local label and candidate mapping	Observed interpretation (working definition)		Key ContSys domains implicated (minimum)	Governance decision and boundary conditions	
*Saatekiri* as referral (candidate: referral)	Mandate transfer (ending an existing mandate)	Responsibility, time, actors, and process and activities			Does this event terminate an existing mandate or start a new commitment? Who is accountable and when? Include nonexamples, such as emergency handover vs planned referral
*Saatekiri* as request (candidate: request)	Joint involvement (addition of a provider while the original mandate is retained)		Responsibility, time, actors, and activities	What responsibilities remain with the originating provider? What is delegated vs transferred? Include nonexamples, such as consultative input without transfer of responsibility	
*Saatekiri* as demand for initial contact (candidate: demand for initial contact)	Episode initiation not tied to prior obligation		Time, process, responsibility, and actors	What constitutes an episode start and its scope? Clarify the boundary with invitation-trigger events, including circumstances in which invitations are treated as referral-like in payer and specification logic	

**Table 5:** Levels of Conceptual Interoperability Model (LCIM) levels and manifestations of saatekiri-type ambiguity across exchange, meaning, context, and governed change over time.

LCIM level	Shared prerequisite (what must be aligned)	Observations from the anchoring case (*saatekiri*)	Governance-relevant artifacts and actions
Level 1: technical interoperability	Connectivity for exchange	Exchange can scale despite divergent event meanings.	Treat connectivity as a baseline only and do not infer shared meaning from connectivity
Level 2: syntactic interoperability	Common message structures and event triggers	A single label can be encoded as a trigger, while different domains intend different events.	Decide on and version the event meaning before encoding it in guidance or specifications
Level 3: semantic interoperability	Shared concept references for exchanged content	Contextual mapping shows conflation of distinct Continuity of Care System of Concepts, and synonyms and notes make mismatches visible.	Maintain a governed concept space and keep notes and mappings versioned and traceable
Level 4: pragmatic interoperability	Shared intent and use context	*Saatekiri* is used as a mandate transfer, joint involvement, or episode initiation.	Maintain an event-meaning register with scope, boundary cases, and nonexamples and link it to policy and payer rules
Level 5: dynamic interoperability	Shared state-change semantics over time	Ambiguity centers on mandate and responsibility movement, episode boundaries, and handover effects.	Establish explicit mandate and responsibility transition rules and maintain change control across policy, payer rules, and implementation guidance
Level 6: conceptual interoperability	Shared aims and assumptions	Event-based exchange presupposes a single cross-domain meaning, and *saatekiri* reveals competing assumption	Establish event-meaning governance, including decision rights, stewardship, conflict resolution, and sustained maintenance

### Early Indications of Use

Adoption outcomes in operational systems have not been evaluated. Related work within the same research program has applied ContSys concepts to structure obligation movement over time in a legal source relevant to care collaboration [35].

### Answer to the Research Question

A familiar referral artifact label (*saatekiri*) carries incompatible assumptions about mandate and responsibility movement, which surface across clinical work, organizational mandate arrangements, payer and entitlement rules, and health information exchange specifications. Therefore, it cannot function as a stable “event” for event-based exchange without an explicit, maintained event-meaning decision.

ContSys translation outputs—interpreted via LCIM and GCM—make these mismatches visible and provide a shared conceptual grammar for turning them into durable governance artifacts (eg, an event-meaning register linked to policy, payer rules, and implementation guidance).

## Discussion

### Principal Findings

This national implementation suggests that adopting a foundational conceptual standard, such as ISO 13940 (ContSys), requires more than mere linguistic translation. Contextualization—systematically connecting concepts to local terminology and legislation while documenting mismatches—was central to stakeholder engagement and to making the standard usable as a conceptual infrastructure.

### Country-Specific Notes as a Governance Mechanism

Country-specific notes served as a mechanism to capture local scope differences and contextual use without adapting the underlying concept system. This supported local understanding while preserving conceptual fidelity.

ContSys deliberately uses neutral language, and stakeholders often begin with familiar local terms that do not align precisely with the underlying conceptual definitions. In *saatekiri*-type discussions, treating a familiar label as a stable proxy for meaning obscured crucial distinctions (mandate transfer vs joint involvement vs episode initiation), so meaning-first, concept-driven alignment was required—even when the resulting formulation felt less natural in everyday language.

### Where Conceptual Gaps Become Critical for Event-Based Exchange

The main barrier observed in contextual mapping was conceptual or semantic rather than technical: familiar labels and local documentation can collapse distinct care process meanings, especially when mandate and responsibility must be explicitly maintained over time. People can often compensate through tacit coordination, but this people-based conceptual alignment does not scale. Human stewards cannot communicate and resolve meaning fast enough as ecosystems grow. Therefore, event-oriented specifications require an explicit, shared, and maintained concept space— a formally documented set of event meanings that can be referenced, versioned, and enforced consistently across clinical operations, organizational accountability, payer rules, and implementation guidance.

LCIM helps locate the issue above the level of interface agreement: exchanging messages does not guarantee shared meaning. GCM adds the evolution constraint: even once a meaning is agreed upon, it will drift unless there is sustained stewardship—clear ownership, decision rights, resourcing, and change control—linking policy and regulation, reimbursement logic, and specifications.

### Implications for Interoperability Governance

Observed discrepancies raise a governance question: should care process event meanings be treated as shared infrastructure, and if so, what stewardship arrangements make them durable?

This study does not claim operational adoption of ContSys as a domain-spanning concept system. Rather, the translation and contextualization work supports a practical hypothesis: ContSys can function as a shared conceptual grammar for event meanings only when there is an explicit coordination model for decision rights, resourcing, conflict resolution, and change control.

Practically, this shifts interoperability work from reliance on familiar artifact labels and tacit coordination toward explicit, cross-domain meaning decisions recorded and maintained across policy, payer rules, and implementation guidance. This is consistent with evidence that unresolved, conflicting stakeholder perspectives during guideline and specification development are a recurrent source of implementation failure and rework [42]. For requirements and specification work, this implies a sequencing rule: first agree on and version the event meaning (Table 4), and only then encode it in profiles and implementation guides, thereby reducing semantic fragmentation and rework arising from incompatible assumptions.

### What Must Be Governed?

For the anchoring case, governance must produce and maintain a pathway-referenced decision indicating which event meaning a given referral artifact label denotes, together with explicit boundary conditions and nonexamples.

Minimum governable artifacts include (1) an event-meaning register (including a definition, scope, and boundary cases); (2) a decision record (including the rationale and endorsing stakeholder domains); and (3) a change control path that links updates in legislation, reimbursement rules, and implementation guidance (eg, profiles and implementation guides) to the maintained event meaning.

More broadly, positioning ContSys as a cross-domain conceptual infrastructure implies a sustained capability to coordinate meanings across policy and regulation, payer logic, and specifications and to manage evolution as service models, rules, and architectures change.

### Limitations

The intervention was limited to translation and contextual mapping and did not include the implementation or testing of computable event definitions in operational systems. Therefore, downstream operational metrics (eg, time to agreement in specification work, interface rework, error reduction, or data quality improvement) were not evaluated. The study also did not analyze correspondence between ContSys concepts and concrete data-exchange message standards (profiles, implementation guides, or message schemas). Consequently, we cannot claim that the candidate event meanings identified here are already encoded consistently in exchange specifications or systems. Evaluating such alignment is a topic for future work.

Several factors may confound the interpretation of discrepancy patterns. Stakeholder participation varied by domain and role, and documentation heterogeneity (differences in legal, organizational, payer, and implementation texts) may bias which mismatches were most visible during mapping. Mapping outputs are comprehensive for the published standard but not exhaustive for all national care process artifacts, and the effects of translation choices vs mapping discussions cannot be fully separated.

Finally, invitation-trigger boundary cases are presented as governance-relevant ambiguities based on observed documentation and discussion patterns. They were not validated through downstream testing in operational specifications or systems.

### Conclusions

We translated and contextualized ISO 13940:2015 (ContSys) as a national standard in Estonia and documented implementation outputs that make local meaning mismatches explicit without redefining core concepts (Tables 1 and 2).

The study indicates that the ambiguity of familiar care process artifact labels can reflect deeper cross-domain disagreement about mandate, responsibility, and episode boundaries. In event-oriented specifications, this becomes a governance risk unless a single event meaning is explicitly decided, recorded, and maintained across policy, payer rules, and implementation guidance (Table 4).

The main implication is governance focused: jurisdictions pursuing event-based exchange should treat event meanings as shared semantic infrastructure and establish stewardship arrangements (decision rights, resourcing, conflict resolution, and change control) that keep policy, reimbursement, and specifications mutually coherent as they evolve.

Future work should extend mapping to additional domains and evaluate how event-meaning governance affects specification effort and operational outcomes in applied settings.

## Abbreviations

ContSys: System of Concepts to Support Continuity of Care
EE NOTE: Estonia-specific note (country-specific note in the national publication)
GCM: Generic Component Model
ISO: International Organization for Standardization
LCIM: Levels of Conceptual Interoperability Model

## Data Availability

The primary output is the national standard publication, available via the national standards catalog (access conditions are defined by the body of standards). Intermediate project artifacts (eg, mapping spreadsheets, working notes, and review materials) are not publicly available with this submission due to third-party rights and copyright constraints. Curated, nonidentifying extracts may be shared upon reasonable request, subject to applicable third-party rights.

## References

[1] Tabari P, Costagliola G, De Rosa M, Boeker M (2024). State-of-the-art Fast Healthcare Interoperability Resources (FHIR)-based data model and structure implementations: systematic scoping review. JMIR Med Inform.

[2] Digital agenda 2030. Ministry of Economic Affairs and Communications, Republic of Estonia.

[3] eHN guideline on patient summary. European Commission.

[4] European Health Data Space Regulation (EHDS). European Commission.

[5] (2018). Continuity and coordination of care: a practice brief to support implementation of the WHO Framework on integrated people-centred health services. World Health Organization.

[6] Haggerty JL, Reid RJ, Freeman GK, Starfield BH, Adair CE, McKendry R (2003). Continuity of care: a multidisciplinary review. BMJ.

[7] Santos MT, Halberstadt BM, Trindade CR, Lima MA, Aued GK (2022). Continuity and coordination of care: conceptual interface and nurses' contributions [Article in English, Portuguese]. Rev Esc Enferm USP.

[8] New generation health information system (upTIS). TEHIK.

[9] (2021). Eesti e-Tervise valitsemisraamistik. Sotsiaalministeerium.

[10] (2022). The development of the Estonian Health System Performance Assessment framework. Organisation for Economic Co-operation and Development.

[11] Metsallik J, Draheim D, Sabic Z, Novak T, Ross P (2024). Assessing opportunities and barriers to improving the secondary use of health care data at the national level: multicase study in the Kingdom of Saudi Arabia and Estonia. J Med Internet Res.

[12] Bertl M, Kankainen KJ, Piho G, Draheim D, Ross P (2023). Evaluation of data quality in the Estonian national health information system for digital decision support. Proceedings of the 3rd International Workshop on Health Data.

[13] Metsallik J, Ross P, Draheim D, Piho G (2018). Ten years of the e-health system in Estonia. Proceedings of the 3rd International Workshop on (Meta)Modelling for Healthcare Systems.

[14] Tiik M (2012). Access rights and organizational management in implementation of Estonian electronic health record system [PhD thesis]. Tallinn University of Technology.

[15] Gilreath CT (1992). Harmonization of terminology – an overview of principles. Int Classif.

[16] (2007). ISO 860:2007: terminology work — harmonization of concepts and terms. International Organization for Standardization.

[17] Tolk A, Muguira JA (2003). The levels of conceptual interoperability model. Proceedings of the 2003 Fall Simulation Interoperability Workshop.

[18] (2015). ISO 13940:2015: health informatics — system of concepts to support continuity of care. International Organization for Standardization.

[19] Kankainen K, Bellatreche L, Chernishev G, Corral A, Ouchani S, Vain J (2021). Usages of the ContSys standard: a position paper. Advances in Model and Data Engineering in the Digitalization Era.

[20] (2013). Towards concurrent use of ContSys, 13606, and HISA. CEN Technical Committee.

[21] Mennerat F, Booth N (2002). Concepts underlying continuity of care - the system of concepts described in ENV 13940a. J Innov Health Inform.

[22] (2021). ISO/TS 22272:2021(en): health informatics - methodology for analysis of business and information needs of health enterprises to support standards based architectures. International Organization for Standardization.

[23] Das S, Hussey P (2021). ContSOnto: a formal ontology for continuity of care. Stud Health Technol Inform.

[24] ISO/PAS 24305:2025: health informatics — guidelines for implementation of HL7 FHIR based on ISO 13940:2015, ISO 13606-1:2019 and ISO 13606-3:2019. International Organization for Standardization.

[25] (2001). Förslag till Svensk version av begreppssystem för kontinuitet i vården: översättning av Europeisk förstandard ENV 13940. Socialstyrelsen.

[26] Klein G (2016). Vägen till Strukturerad Information i Vårde: Delrapport inom Vinnova projektet 3H3R.

[27] Blobel B, Oemig F, Ruotsalainen P (2018). Data modeling challenges of advanced interoperability. Building Continents of Knowledge in Oceans of Data: The Future of Co-Created eHealth.

[28] Blobel B, Ruotsalainen P, Oemig F (2020). Why interoperability at data level is not sufficient for enabling pHealth?. Stud Health Technol Inform.

[29] Brochhausen M, Bost SJ, Singh N, Brochhausen C, Blobel B (2021). Understanding the gap between information models and realism-based ontologies using the generic component model. Stud Health Technol Inform.

[30] Blobel B, Ruotsalainen P, Oemig F, Giacomini M, Sottile PA, Endsleff F (2023). Principles and standards for designing and managing integrable and interoperable transformed health ecosystems. J Pers Med.

[31] Oemig F, Blobel B (2022). Modeling digital health systems to foster interoperability. Front Med (Lausanne).

[32] (2021). ISO 23903:2021: health informatics — interoperability and integration reference architecture — model and framework. International Organization for Standardization.

[33] Blobel B (2019). Challenges and solutions for designing and managing pHealth ecosystems. Front Med (Lausanne).

[34] Kankainen KJ, Erm R, Metsallik J, Piho G, Ross P (2025). Experiences translating and introducing ISO 13940 ContSys in Estonia: challenges and perspectives. Stud Health Technol Inform.

[35] Kankainen KJ, Erm R, Metsallik J, Piho G, Ross P (2025). Method for ContSys-based semantic annotation of the Estonian law of obligations. Stud Health Technol Inform.

[36] Tolk A, Harper A, Mustafee N (2021). Hybrid models as transdisciplinary research enablers. Eur J Oper Res.

[37] Tolk A, Diallo SY, Turnitsa CD (2007). Applying the levels of conceptual interoperability model in support of integratability, interoperability, and composability for system-of-systems engineering. J Syst Cybern Informatics.

[38] Blobel B, Oemig F, Ruotsalainen P, Lopez DM (2022). Transformation of health and social care systems-an interdisciplinary approach toward a foundational architecture. Front Med (Lausanne).

[39] (2002). Law of Obligations Act. Riigi Teataja.

[40] E-tervise tooted. Tervisekassa.

[41] Ross P, Metsallik J, Kankainen KJ, Bossenko I, Mäe C, Maasik M (2023). Health Sense: development of a universal data model and a standard for continuity of treatment paths based on international standards of new generation health information systems. Zenodo.

[42] Michaels JA, Maheswaran R (2023). Conflicting perspectives during guidelines development are an important source of implementation failure. Health Policy.

